# The Impact of Bioceramic Scaffolds on Bone Regeneration in Preclinical In Vivo Studies: A Systematic Review

**DOI:** 10.3390/ma13071500

**Published:** 2020-03-25

**Authors:** Giulia Brunello, Sourav Panda, Lucia Schiavon, Stefano Sivolella, Lisa Biasetto, Massimo Del Fabbro

**Affiliations:** 1 Department of Management and Engineering, University of Padova, Stradella San Nicola 3, 36100 Vicenza Italy; giulia-bru@libero.it (G.B.); lisa.biasetto@unipd.it (L.B.); 2 Section of Dentistry, Department of Neurosciences, University of Padova, Via Giustiniani 2, 35128 Padova, Italy; luciaschiavon.08@gmail.com (L.S.); stefano.sivolella@unipd.it (S.S.); 3 Department of Biomedical, Surgical and Dental Sciences, Università degli Studi di Milano, Via Commenda 10, 20122 Milan, Italy; sourav.panda@unimi.it; 4 Department of Periodontics and Oral Implantology, Institute of Dental Sciences, Siksha O Anusandhan University, Bhubaneswar, 751003 Odisha, India; 5 Dental Clinic, I.R.C.C.S. Orthopedic Institute Galeazzi, Via Galeazzi 4, 20161 Milan, Italy

**Keywords:** animal study, bioceramic, bone grafting, critical-sized bone defect, scaffold

## Abstract

Bioceramic scaffolds are appealing for alveolar bone regeneration, because they are emerging as promising alternatives to autogenous and heterogenous bone grafts. The aim of this systematic review is to answer to the focal question: in critical-sized bone defects in experimental animal models, does the use of a bioceramic scaffolds improve new bone formation, compared with leaving the empty defect without grafting materials or using autogenous bone or deproteinized bovine-derived bone substitutes? Electronic databases were searched using specific search terms. A hand search was also undertaken. Only randomized and controlled studies in the English language, published in peer-reviewed journals between 2013 and 2018, using critical-sized bone defect models in non-medically compromised animals, were considered. Risk of bias assessment was performed using the SYRCLE tool. A meta-analysis was planned to synthesize the evidence, if possible. Thirteen studies reporting on small animal models (six studies on rats and seven on rabbits) were included. The calvarial bone defect was the most common experimental site. The empty defect was used as the only control in all studies except one. In all studies the bioceramic materials demonstrated a trend for better outcomes compared to an empty control. Due to heterogeneity in protocols and outcomes among the included studies, no meta-analysis could be performed. Bioceramics can be considered promising grafting materials, though further evidence is needed.

## 1. Introduction

One of the major challenges in dentistry, and in maxillofacial and orthopedic surgery, still remains to be the reconstruction of extensive bone defects [1,2]. The ideal bone substitute should be biocompatible, osteoconductive, and resorbable, and thereby replaced by newly formed bone, while maintaining adequate mechanical strength and structural support in the meantime, especially in load-bearing applications [3,4,5].

Ceramic materials have been successfully used for the reconstruction of bone tissue defects [6,7]. The term bioceramics comprises a broad range of biocompatible inorganic non-metallic materials, characterized by a crystal structure, high melting point, electrical resistivity, and corrosion resistance [8,9]. These features make them suitable for a variety of applications, including oral and maxillofacial surgery, periodontal treatments, and orthopedics [8]. However, one of the major drawbacks of ceramic scaffolds consists of their brittle behavior, which has restricted their use mainly to non-load-bearing applications [10].

Among various bioceramics, calcium phosphates, such as hydroxyapatite (HA) and tricalcium phosphate (TCP), are commonly used bone grafting materials due to their resemblance to the bone mineral phase [10,11]. Besides calcium phosphate ceramics, more recently a new class of biomaterials, known as silicate bioceramics, have received significant attention for hard tissue regeneration [12,13,14,15]. 

The variety in chemical composition of bioceramics contributes to their adjustable mechanical features, bioactivity, and degradation rate. Another strategy to produce scaffolds with tailored mechanical properties and resorbability, based on application needs, consists of the development of composite materials, containing bioceramics and polymers in different ratios [16,17]. To improve the performances of bioceramic scaffolds, the incorporation of growth factors stimulating osteogenesis and angiogenesis has been described [18,19]. Moreover, bone scaffolds could act as stem cell carriers for accelerating bone repair [20].

In order to test bone substitute materials, preclinical in vivo studies in clinically relevant animal models are a fundamental step in translational research [21,22]. Various experimental approaches have been proposed, including the “critical-sized defect” (CSD) model [23,24]. An intrabony defect of critical dimensions is not expected, by definition, to heal spontaneously within the lifetime of the animal [25,26]. CSD models have been described for many kind of animal models. Among them, the use of rabbits and rats offers the advantages of easy handling and reduced experimental costs and timing. Despite the higher similarity to human bone (e.g., anatomy, biomechanics), the use of larger-sized animals, such as dogs or pigs, is limited due to high experimental costs, more demanding management, the need for long follow-ups, and ethical concerns [21,27,28,29].

In order to assess new bone formation, several methods have been utilized, such as histological and histomorphometric analyses, gene expression analysis, and radiographic evaluations. Micro-CT analysis has been recently introduced as a complementary non-destructive approach to assess bone healing [30,31]. It does not require the sectioning of the sample, which might affect the three-dimensional anisotropic information of bone architecture [30].

There are many reviews about different kinds of ceramic scaffolds for bone tissue regeneration, mainly focusing on biomaterial properties and production methods [6,7,10,12,14]. However, although preclinical in vivo studies in clinically relevant animal models represent a key aspect of translational research, there is no systematic review investigating the effects of bioceramic scaffolds on bone formation in CSD in experimental animal models, compared with the blood clot alone or with widely investigated materials, such as autogenous bone or deproteinized bovine-derived bone mineral (DBBM). 

Hence, the aim of this systematic review was to investigate the results of the application of bioceramic scaffolds in terms of bone regeneration in the treatment of CSDs in vivo in comparison with leaving the empty defect without grafting materials or with the use of autogenous bone or DBBM. The quality of the available studies was also assessed. 

## 2. Materials and Methods 

The protocol for this review was registered with the international prospective register of systematic reviews (PROSPERO) with registration n. CRD42019139963.

### 2.1. Focal Question

The present systematic review was conducted in accordance with the Preferred Reporting Items for Systematic Reviews and Meta-Analyses (PRISMA) guidelines [32]. 

The focused “PICO” (population, intervention, comparison, outcome) question addressed was the following: in bone defects in experimental animal models, does the use of a bioceramic scaffold improve new bone formation, compared with leaving the empty defect without grafting materials or using autogenous bone or deproteinized bovine-derived bone substitutes?

### 2.2. Eligibility Criteria

#### 2.2.1. Inclusion Criteria

- Publication written in English.
- Randomized or non-randomized controlled animal experimental studies with at least two study groups and at least 6 animals per group.
- Use of experimental critical-sized bone defect (CSD) in non-medically compromised animals.

#### 2.2.2. Exclusion Criteria

- In vitro studies, clinical studies, reviews, meta-analyses, conference proceedings, book chapters.
- Animal studies reporting ectopic models (e.g., subcutaneous).
- Absence of an empty defect and/or autogenous bone and/or deproteinized bovine-derived bone substitutes control group.
- Treatment of periodontal defects.
- Studies using scaffolds loaded with chemotherapeutic agents, anti-inflammatory drugs, antibiotics.

Studies using scaffolds loaded with drugs/stem cells/substances affecting bone metabolism were not excluded. Table 1 summarizes the dimensions of the critical-sized bone defects in different animal models.

Tooth extraction socket model was not considered a critical-sized bone defect model.

### 2.3. Search Strategy, Screening Method, and Data Extraction

The protocol for this review was registered with the international prospective register of systematic reviews (PROSPERO) with registration number CRD42019139963. The MEDLINE (PubMed) online library and the Web of Science (WoS) database were searched on 21th November 2018. The search was limited to studies published between January 2013 and November 2018. The time-frame was selected considering the recent advancements in biomaterial production, such as the rise of additive manufacturing technologies.

For the identification of studies to analyse for the present systematic review, detailed search strategies were developed for both databases, using a combination of the following keywords: “animal,” “bioceramic,” “bone,” “bone defect,” “bone regeneration,” “grafting,” and “in vivo.” Details of the search strategy are provided in the Supplementary Materials, Table S1.

A 2-stage screening was carried out. The screening of the titles and of the abstracts was performed in duplicate and independently by two reviewers (G.B. and L.S.). Full texts of all eligible articles were obtained and reviewed independently by the same two reviewers (G.B. and L.S.). For each study, relevant data were extracted and recorded on a previously designed data collection form. The final inclusion was based on the aforementioned eligibility criteria. Reasons for exclusion were also entered. Cohen’s kappa statistic was calculated at both the stages, titles/abstracts and full texts, to measure the level of agreement between the two reviewers. In case of disagreement, when a consensus between the two reviewers was not reached after discussion, a third experienced reviewer (M.D.F.) was consulted. 

### 2.4. Outcome Measures

#### 2.4.1. Primary Outcomes

New bone formation can be measured with different techniques (e.g., histomorphometric analysis, radiographic analysis like computed tomography (CT), micro-CT, standard radiographs); residual biomaterial.

#### 2.4.2. Secondary Outcomes

Any complications and adverse events related to the biomaterials used.

Scaffold production and characterization were also investigated.

### 2.5. Quality Assessment and Risk of Bias Analysis

The quality of the studies was assessed independently by two reviewers (S.P. and G.B.), based on the ARRIVE (Animals in Research: Reporting In Vivo Experiments) guidelines [47]. The items considered were the following: ethical statement, experimental procedures, experimental animals, randomization, allocation concealment, sample size calculation, completeness of information, blinding of the evaluator, and financial conflict of interest.

The risk-of bias of the studies was assessed by using the SYRCLE tool [48], evaluating 10 items. All items could be judged as yes/no/unclear. Studies were considered at high risk of bias if at least two items were judged as “no.” Studies were judged as low risk of bias if at least 7 items were judged as “yes” and no item was judged as “no.” In other cases the studies were considered at medium risk of bias.

### 2.6. Data Synthesis and Statistical Analysis

Due to the heterogeneity in study protocols, biomaterials used, methods for assessing the outcomes, outcome measures, and follow-up duration, no meta-analysis could be performed. Only qualitative data extracted from each study were synthesized in analytic tables.

## 3. Results

### 3.1. Study Selection

**Figure 1:**
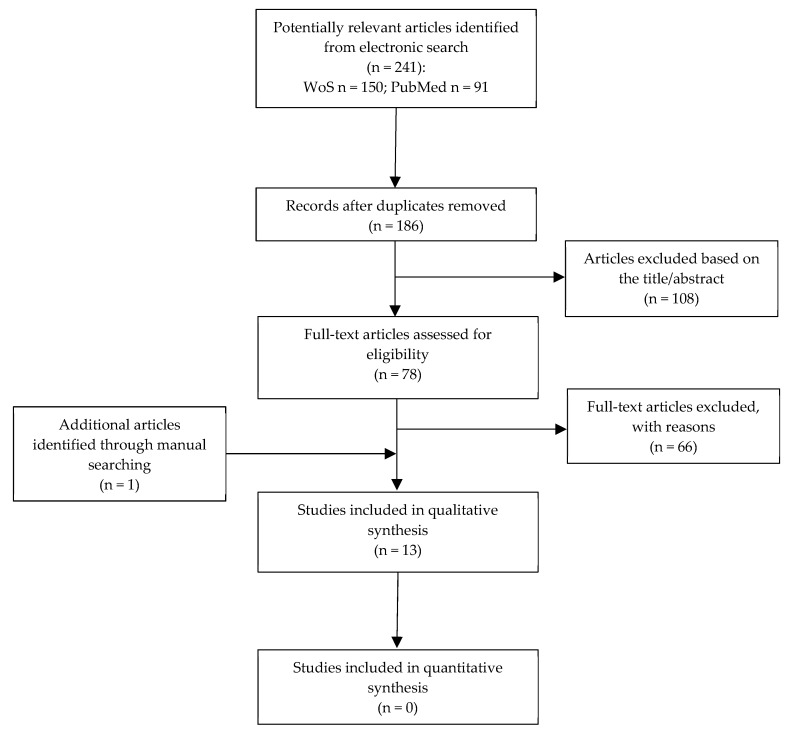
Flowchart of the article selection procedure.

Only qualitative data extracted from each study were used in analytic tables. A total of 186 articles were reviewed. After title/abstract screening, 78 articles were included as relevant for the purpose of the present systematic review. Following the final screening of full texts, 12 articles fulfilled the inclusion criteria and 66 papers were excluded. The reasons for exclusion are summarized in Table 2. The kappa values for inter-reviewer agreement were 0.91 and 0.90 for title/abstract selection and for full-text articles, respectively, thereby indicating almost perfect agreement. An additional article identified by handsearching was also included. Flow diagram of search results is shown in Figure 1: the number of articles for quantitative analysis was equal to zero; for this reason data were only qualitatively discussed.

### 3.2. Study Characteristics 

Only qualitative data extracted from each study were synthesized in analytic tables. In seven of the 13 included studies, New Zealand rabbits were used [16,115,116,117,118,119,120], while six studies were conducted in rats, of which three used the Sprague–Dawley strain [17,121,122], two the Wistar strain [123,124] and one the Lewis strain [125]. The calvarial critical-sized defect was the most used model for assessing new bone formation (Table 3).

Two studies used bone marrow-derived mesenchymal stem cells (BMSCs) of different origins [17,115,120]. Interestingly, in none of those studies was the use of resorbable or non-resorbable membranes reported. Histological evaluation was the most frequent evaluation method (n = 13) to assess bone healing, followed by histomorphometric analysis (n = 6); radiographic evaluation (n = 4), micro-computed tomography analysis (n = 4); and other methods, less represented, including real-time polymerase chain reaction (real-time PCR), Western blot, immunofluorescence, immunohistochemistry, scanning electron microscopy (SEM), and multi slice spiral computer tomography (MSCT). Follow-ups varied between two and 18 weeks. A single observation time was reported in two out of 13 studies [120,121], while the other studies had multiple observation times. 

As the chemical composition and processing technology are considered key factors for determining the properties of the scaffolds, they were analyzed and summarized in Table 4. 

A variety of production methods were reported, leading to the manufacturing of scaffolds with different compositions and morphologies, from 3D bone structures to particles of smaller dimensions, such as the microspheres employed in Xu et al. [117]. The definition of bone scaffold was not limited to 3D bone structures, but it was here used to describe a matrix allowing and stimulating cell attachment and proliferation on its surfaces. Interestingly, addictive manufacturing technologies, which present the main advantage of producing customized scaffolds tailored to the specific critical-size bone defect [11], were utilized in two studies [116,122]. 

As regards 3D bone structures, which could not only promote new bone formation, but could potentially be submitted to a mechanical load before the bone healing process is complete, no mechanical characterization was reported in all included studies, but one [116]. In Shao et al. [116], it was found that the dilute Mg doping and/or two-step sintering schedule was particularly beneficial for enhancing the mechanical strength of CaSi scaffolds, as reported in Table 4.

Even though porosity and pore size are considered key parameters influencing the biological properties of biomaterials, as a porous structure provides an ideal environment for bone tissue ingrowth and repair, only in four studies was the porosity evaluated, with values ranging between 53 and 93 vol.% (see Table 4) and pore size ranging between 100 and 500 µm.

The chemical dissolution of the scaffold should be evaluated, as the mechanical integrity of the scaffold could be compromised during the healing time. Moreover, the release of some components might participate in human metabolism, thereby affecting bone formation. Only in two papers was the in vitro resorbability assessed (see Table 4) [17,118]. In addition, in Zong et al. [17] the scaffolds were implanted intramuscularly into rats to examine the in vivo degradation with results consistent with the in vitro findings.

For simplicity, the included studies are presented based on the animal model. 

#### 3.2.1. Studies in Rabbits—Main Features

The characteristics and the main results of the studies in rabbits are summarized in Table 5. Notably, all the included studies reported uneventful healing outcomes and no relevant adverse reactions. Two studies reported on the use of polyether-ether-ketone/odontogenic biphasic bioceramic composites (PEEK-BBC) prepared via calcination for the treatment of mandibular bone defects [16,115]. Porous PEEK-BBC composites were found to promote bone healing in vivo, potentially via the upregulation of bone morphogenetic protein-2 (BMP-2), as suggested by the higher mRNA and protein expression levels of BMP-2 in the presence of PEEK-BBC composites, than in bone defects left empty [16]. Moreover, when vascular endothelial growth factor (VEGF) was encapsulated into PEEK-BBC composites, a relative upregulation of VEGF at 8 and 16 weeks of healing was observed compared to jaw defects left empty [115]. However, the specific effect of the exogenous VEGF, itself, encapsulated in the PEEK-BBC composites, could not be determined, due to the absence [115] of a control group treated with PEEK-BBC alone.

**Figure 2:**
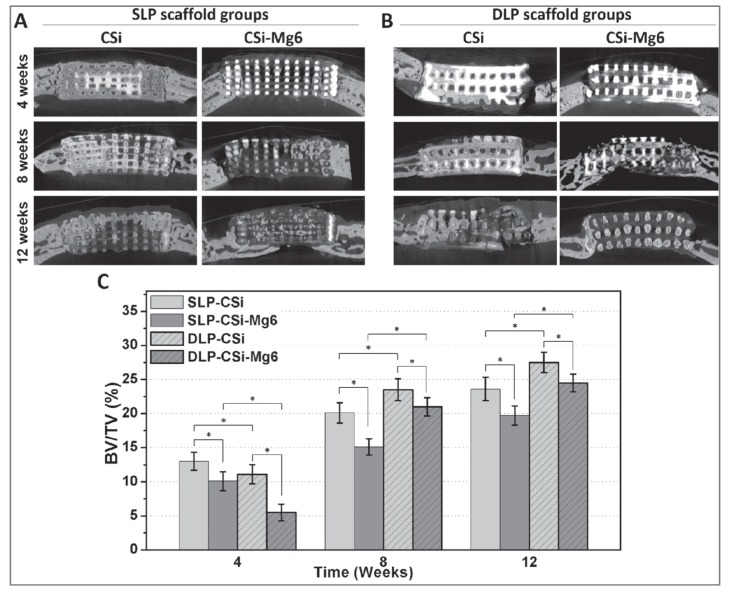
Osteogenesis of the ceramic scaffolds in vivo. (**A**) The cross-sectional images of implanted ceramic scaffolds of CaSi and CaSi–Mg6 with single layer pore morphology by microCT scanning after 4, 8, and 12 weeks, respectively. (**B**) The cross-sectional images of implanted ceramic scaffolds of CaSi and CaSi–Mg6 with double layer pore morphology by microCT scanning after 4, 8, and 12 weeks, respectively. (**C**) Morphometric analysis of the volume of the newly formed bone (BV/TV) in the skull defect area at 4, 8, and 12 weeks with single layer pore morphology and double layer pore morphology, respectively. (*p < 0.05) [116].

In two studies the same calvaria bone defect model was used to assess the osteoconductive properties of different calcium phosphate and silica-based bioceramics [116,117]. In Shao et al., the in vivo behaviors of 3D-printed pure calcium silicate (CaSi) and dilute Mg-doped CaSi (CaSi–Mg6) scaffolds, characterized by different side-wall pore architectures depending on the deposition mode, were investigated [116]. Single-layer printing (SLP) scaffolds, featured by smaller layer thickness and interconnection size, exhibited a higher osteogenic capacity than double-layer printing (DLP) scaffolds in early phases (4 weeks). DLP scaffolds showed higher osteoconduction in later healing stages. Twelve months postoperatively, the highest percentage of new bone was observed in the group treated with CaSi scaffolds with double layer pore morphology (~26%), followed by DLP CaSi–Mg6 (~23%). Even though DLP CaSi scaffolds promoted new bone formation to a greater extent, Mg doping considerably enhanced the mechanical properties of the scaffolds, which might be required in particular clinical situations. Details are provided in Figure 2.

Dual-shell microspheres, composed of layers of slowly degraded β-TCP and rapidly degraded β-CaSi, displayed different bone regeneration patterns depending on the distribution of the materials within the dual-shell architectures [117]. Microspheres characterized by a core and an external layer of CaSi, separated by an intermediate β-TCP layer (CaSi@CaP@CaSi), showed superior performances in vivo, which might be due to the quick degradation of the external CaSi layer leading to an increased local silicon ion concentration. Interestingly, using micro-CT data from 12 weeks of healing, CaSi@CaP@CaSi microspheres [117] showed a bone volume/total defect volume ratio (BV/TV) of approximately 20% like SLP CaSi–Mg6 scaffolds [116], while the other 3D-printed scaffolds investigated in Shao et al. [116] exhibited higher values up to 27.5%. 

Calcium sulfate (CS) was utilized only in Li et al. [118], wherein it was incorporated into poly(amino acid) (PAA), to reduce the excessively rapid degradation rate of the former. Two CS/PAA composites containing 50% and 65% (mass fraction) of CS were produced via the in situ melting polymerization method and tested in a femoral bone defect model up to three months. Both the composites displayed good biocompatibility and similar amounts of newly formed bone. However, as in preliminary in vitro evaluations, the granules with higher CS content (65CS/PAA) exhibited a faster degradation rate.

Resorbable biphasic calcium phosphate scaffolds, composed of HA and TCP, tested the remaining two included studies in rabbits [119,120]. In Ezirganlı al. [119], after three months of healing, the amount of newly formed bone was similar in DBBM and bicalcium phosphate groups. However, only DBBM group showed a significantly higher new bone formation compared to the empty group used as the control. Moreover, BMSCs of different origin (i.e., autologous, allogenic, ovine, and canine) seeded on biphasic calcium phosphate scaffolds were found to enhance new bone formation in radial segmental bone defects compared to defects filled with cell-free scaffolds and to untreated ones [120].

#### 3.2.2. Studies in Rats—Main Features

The characteristics and the main findings of the studies in rats are provided in Table 6.

In none of the included studies were adverse reactions to the implanted biomaterials reported. To evaluate the osteogenic potential of the bioceramics in rats, calvarial critical-sized defects were used in all studies but one [125]. 

Porous composite scaffolds, composed of HA and polylactic acid (PLA) and produced with different techniques, were tested in two studies [17,122]. In Zong et al. [17], nano-HA/PLA scaffolds fabricated by a porogen-leaching technique and loaded with BMSCs were able to induce bone formation in vivo. Nevertheless, higher new bone formation was detected in defects grafted with poly(lactic-co-glycolic acid) (PLGA) scaffolds seeded with BMSCs, with average new bone formation of about 50% after 16 weeks of healing, against the approximately 30% found in the group treated with nano-HA/PLA loaded with BMSCs. It was inferred by the authors that the lower degradation of nano-HA/PLA scaffold compared to PLGA matrix could be responsible for its inferior bone-repairing effects. Interestingly, no bone regeneration was observed in defects filled with nano-HA/PLA scaffolds alone. 

**Figure 3:**
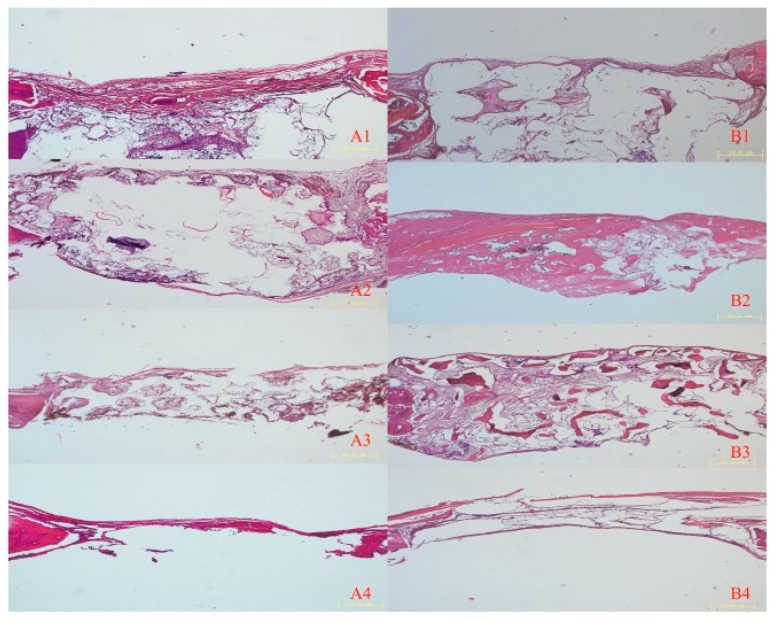
Hematoxylin and eosin images of implanted and control group after four and eight weeks. (**A**) Histological images of implanted (1) PLA/HA, (2) β-TCP, and (3) DBM scaffolds, and (4) the control group four weeks after implantation. (**B**) Histological images of (1) implanted PLA/HA, (2) β-TCP, and (3) DBM scaffolds, and (4) the control group eight weeks after implantation. Scale bars 10 μm [122].

In contrast with these findings, in Zhang et al. [122] highly resorbable three-dimensional (3D) printed PLA/HA scaffolds showed good bone repairing capacity, as confirmed by histological examination (Figure 3). As revealed by micro-CT data, both at four and eight weeks after surgery the highest amount of bone volume per total volume (BV/TV) was found in the defects filled with β-TCP ceramic scaffolds, with values around 50%, followed by PLA/HA scaffolds, and then, by partially demineralized bone matrix (DBM). 

Combining HA with a fibrin sealant (FS) derived from snake venom exerted a beneficial effect on bone healing, compared to HA or fibrin sealant alone, as confirmed by histomorphometric analysis [123]. Six weeks postoperatively, the highest relative volume of new bone was recorded in HA/FS samples (53.66 ± 0.57%), whereas in empty defects, HA and FS groups lower values were registered (i.e., 10.66 ± 0.57%, 20.66 ± 1.15%, and 29.66 ± 1.52%, respectively).

In two papers the osteoconductive capacity of the bioceramic scaffolds was evaluated using 8 mm cylindrical bone defects in a rat’s calvaria [114,124]. Despite the same model being used in these studies, no direct comparison could be drawn due to the different timepoints selected by the authors. 

Silica aerogel-based β-TCP composite was demonstrated [124] to better support new bone formation compared to the mesoporous silica aerogel alone. Interestingly, three months after implantation, most of the aerogel-based β-TCP composite was resorbed and signs of intense bone remodeling and ossification were confirmed by histological observations and immunohistochemistry for Ki-67. At this stage, bone defects filled with silica aerogel alone exhibited bone ossification to a lower extent, whereas only a minimal ossification in the periphery of the defects was detected in the untreated control group. 

PLGA electrospun nanofibers coated with a bioactive silica-based ceramic containing zinc and willemite (Zn_2_SiO_4_) were proved to be promising candidates for bone tissue engineering applications [121]. After 8 weeks of healing, in the defects treated with willemite-coated PLGA scaffolds, the area of reconstructed bone tissue, resulting from quantitative analysis of histologic and multislice spiral-computed tomography data, was found to be approximately 70%, twice the amount of bone detected in the rats receiving PLGA scaffolds with no bioceramic coating. 

Another silica-based ceramic, merwinite [Ca_3_Mg(SiO_4_)_2_], was found to enhance new bone formation in rat femoral defect model to a greater extent than HA ceramics and leaving the defects unfilled [120]. It is likely that the higher in vivo material degradation of merwinite granules compared to HA ones induced a wider and faster osteogenesis, hence confirming the superior bioactive properties of this material. 

### 3.3. Study Quality and Risk of Bias Assessment

A study quality assessment according to the ARRIVE guidelines is shown in Table 7. Scoring criteria are provided in Table S2.

All the studies reported data on ethical statements and provided detailed information about the experimental procedures and outcome evaluation (items 1, 2, 7, respectively). All the studies, except one, gave adequate information about experimental animals (item 3), while the majority of studies lacked complete information regarding allocation concealment (item 5) and blinding of the evaluator (item 8). Only in six studies (46.1%) were animals or defects randomly allocated to different treatment groups (item 4) and no study provided information on the sample size calculation (item 6). Finally, regarding financial conflict of interest and possible role of the funders, approximately half of the studies (53.8%) provided clearly adequate information, whereas in the remaining six studies, the information was unclear/possibly adequate.

Risk of Bias Assessment of the selected studies according to the SYRCLE tool is provided in Table 8. 

## 4. Discussion

The aim of this systematic review was to investigate the role of bioceramic scaffolds in regenerating critical-sized bone defects in experimental animal models, compared with leaving the empty defect without grafting materials or filling the defects with autogenous bone or deproteinized bovine-derived bone substitutes. Overall, the results showed that bioceramic scaffolds better supported new bone formation, compared to untreated empty defects. In general, there was only a limited spontaneous bone regeneration at the site of defects substituted with no material. In none of the included studies were autogenous bone grafts used as controls, whereas, when DBBM was considered, a similar amount of new bone formation was observed in DBBM and bioceramic groups [119].

The most frequent reason for exclusion was the absence of a control group overall, along with the absence of a control group consisting of leaving the defect without any biomaterials and/or filling it with autogenous bone graft and/or DBBM. Eleven papers were not included due to the sizes of the defects, which were not considered of critical dimensions. 

Numerous bioceramic and composite materials for bone regeneration were developed and tested in vivo in CSD. However, the considerable heterogeneity among the selected studies, in terms of scaffold composition, size, and type of the defect and observation time, did not allow cross-study comparisons. Indeed, due to the lack of standardization of these variables across the studies together with the few quantitative data reported, a meta-analysis could not be performed and the generalizability was limited. 

No extensive physico-chemical and mechanical characterization was reported in most of the studies. Regarding the fabrication of the scaffolds, resumed in Table 4, many production technologies were applied, leading to the manufacturing of bioceramic powders within a polymeric matrix or 3D scaffolds. In two studies [119,125], commercially available biomaterials were used. However, for the other produced materials, only lab-scaled processes were investigated. 

Although studies in dogs, minipigs, sheep, and non-human primates, could provide a better insight into new bone formation and scaffold effectiveness thanks to the closer resemblance to the human bone, only studies employing rat and rabbit models were found to satisfy eligibility criteria, and were, therefore, included in the present systematic review [21,27,28,29,126]. Even though after the first step of screening, studies in dogs, sheep, and pigs were included, the full-text analysis revealed that most of these studies did not meet the selection criteria due to a reduced sample size (n < 6 animals per group) or the non-critical dimensions of the bone defects [94,100,102,103,106]. Among the papers included, the most frequently used CSD model was the CSD in rat calvaria, which is one of the most commonly used animal models for evaluating bone healing [23,127].

Despite the high heterogeneity among the studies, bioceramic scaffolds generally showed a remarkable osteoconductive effect. However, it was not possible to determine which bioceramic performed better than the others and in regard to which kind of CSD.

Scaffold architecture is considered a fundamental aspect in tissue engineering. A bone scaffold should present an interconnected porous structure mimicking that of natural bone, thus facilitating cell ingrowth, proliferation, and differentiation, as well as the diffusion of nutrient and the removal of waste products [128]. In the meantime, the scaffold should possess adequate mechanical properties, which are particularly required for load-bearing applications [129]. It has to be stressed that, although these aspects are of primary importance, in most of the articles no comprehensive characterization of the scaffolds was provided and compressive strength values were reported in only one work [116].

An ideal scaffold should also possess an adequate degradation rate, matching the osteogenesis rate occurring in the replaced bone [118,130]. One of the strategies to tailor the biodegradability of a scaffold consists of the development of composite materials, composed of biodegradable polymers and bioceramic particles, added as fillers or as coatings. Therefore, in composites the osteoconductive properties of the bioceramics are combined with the easy processing and faster resorbability of the polymers [17,118,131,132]. Interestingly, in two articles the in vitro degradation of the scaffolds was evaluated [17,118]. In Li et al. [118], two calcium sulfate/poly(amino acid) (CS/PAA) scaffolds, characterized by different CS content, exhibited weight losses of 41.5% and 56.2% after soaking in simulated body fluid (SBF) for 16 weeks, thereby indicating that the relative amount of CS in the composite affected the degradability of the material. In contrast, in Zong et al. [17], after 8 weeks in phosphate buffered saline (PBS), the weight loss of the composite scaffold (i.e., nHAP/PLA) was nearly 10% of its initial weight, while the weight loss rate of PLGA scaffold was much higher, with values around 50%. The authors assumed that the in vivo performances of nHAP/PLA scaffolds in terms of new bone formation were lower than expected due to the low degradation of the scaffold, hampering the regeneration process. 

With regard to the incorporation of bioactive molecules for localized and controlled delivery [18,19], in one study [115] VEGF, a potent angiogenic factor, was successfully encapsulated within a PEEK/biphasic bioceramic composite scaffold and found to facilitate the vascular remodeling in vivo. 

Furthermore, bone scaffolds were used as stem cell carriers for accelerating and promoting bone repair in two articles [17,120]. In particular, BMSCs, pluripotent mesenchymal stem cells with the proven ability to differentiate into different cell lineages, including osteoblasts [133,134,135], were used in both studies. The combination of BMSCs and HA/TCP scaffolds for the treatment of rabbit segmental radial bone defects showed increased quantity of newly formed compared to the bioceramic scaffold alone [120]. Moreover, a higher bone-repairing effect was exhibited by nano HA/PLA scaffolds seeded with BMSCs than by the composite scaffolds alone in rat calvarial CSD model [17]. These findings are in agreement with what has been reported in other studies, in which bone regeneration was aided by the addition of BMSCs seeded onto the scaffolds before implantation [136,137].

## 5. Conclusions

In conclusion, several bioceramic scaffolds were demonstrated to be osteoconductive in a variety of animal models, showing better results than leaving the bone defects with no grafting material. It was not possible to compare the investigated scaffolds with autogenous bone, and only in one study was DBBM evaluated, showing similar behavior in vivo. The results also indicated that composite materials, comprising bioceramic particles and polymers, could be promising candidates as bone substitutes. Bioceramic scaffolds should therefore be applied in the repair of bone defects on a regular basis, in order to promote bone tissue healing. Regarding the use of stem cells or growth factors, there is still scarce, though promising evidence that the addition of mesenchymal stem cells or VEGF to the scaffolds further enhances bone regeneration in preclinical in vivo studies.

However, due to the high variability among the studies with regard to the compositions of the biomaterials, the production methods, the type and dimensions of the bone defects used, and the follow-up duration, no conclusive statements about the clinical effectiveness of bioceramic scaffolds for bone regeneration can be made. In the future, in vivo animal models should be designed following standardized parameters (i.e., adoption of critical-sized defects, empty control group, and quantitative measurements for bone formation), in order to allow the comparison of findings, thereby favoring the advancement of knowledge in this fast-growing area of research. Moreover, further studies are needed in order to determine the optimal evaluation times for each CSD in different animal models.

## Figures and Tables

**Table 1:** Definition of critical-sized bone defect (CSD).

Animal	Defect Site	Dimension of CSD	References
Mouse	Calvaria	4 mm diameter	[33]
	Segmental long-bone defect	Radius: 4 mm Femur: 5 mm	[34]
Rat	Calvaria	Unilateral/central:8 mm diameter; bilateral: 5 mm diameter	[23]
	Cylindric defect	Femur: 2 mm in diameter and 3 mm in length	[35]
	Segmental long-bone defect	Radius: 1 cm diameter	[36]
	Mandible	4 mm diameter	[37]
Rabbit	Calvaria	Four defects: 8 mm diameter; unilateral defect: 15 mm diameter; bilateral defect: 11 mm diameter	[38]
	Segmental long-bone defect	Radius: defect > 1.4 cm involving periosteum	[39]
	Cylindric defect	Femur: 6 mm in diameter and 5 mm in length; tibiae: 6 mm diameter	[40] (femur) [41] (tibiae)
	Mandible	5 mm diameter	[42]
Pig	Segmental long-bone defect	Femur: 7.6 cm; tibiae: 2 cm; radius: 2.5–3 cm; ulna: 2 cm	[34,36]
Sheep	Calvaria	22 mm in diameter	[43,44]
	Segmental long-bone defect	Femur: 2.5 cm; tibiae: 3–3.5 cm	[34]
Dog	Calvaria	2 cm	[45]
	Segmental long-bone defect	Femur: 2.1–7 cm; radius: 0.3–2.5 cm; ulna: 2–2.5 cm	[34]
	Segmental mandibular defect	50 mm (in presence of periosteum); 15 mm (in absence of periosteum)	[46]

**Table 2:** Main reasons for exclusion after full-text screening.

Main Reason for Exclusion	No.	References
Language	3	[49,50,51]
In vitro study	2	[52,53]
Ectopic bone formation model	4	[54,55,56,57]
Use of compromised animals	4	[58,59,60,61]
Absence of a control group	13	[62,63,64,65,66,67,68,69,70,71,72,73,74]
Control group other than empty defect and/or autogenous bone and/or deproteinized bovine-derived bone	20	[75,76,77,78,79,80,81,82,83,84,85,86,87,88,89,90,91,92,93,94]
Unclear sample size	5	[95,96,97,98,99]
Less than 6 animals per each test group	4	[100,101,102,103]
Non-critical size bone defect	11	[104,105,106,107,108,109,110,111,112,113,114]

**Table 3:** Distribution of defect types among the included studies.

Animal	Study Model	Number of Publications	References
Rabbit (n = 7)	Calvarial defect	2	[116,117]
	Dome model (calvaria)	1	[119]
	Cylindrical femoral defect	1	[118]
	Segmental radial defect	1	[120]
	Mandibular square hole	2	[16,115]
Rat (n = 6)	Calvarial defect	5	[17,121,122,123,124]
	Cylindrical femoral defect	1	[125]

**Table 4:** Bone scaffold production method and main properties.

Ref.	Biomaterial(s)	Production Method	Morphology	Porosity (%)	Density (g cm^−3^)	Elastic Modulus (MPa)	Compressive Strength (MPa)	In vitro Resorbability
[115]	PEEK-BBC composite doped with VEGF	HA + β-TCP bioceramic powder derived from extracted teeth, then impregnation in organic foam to prepare PEEK/BBC composite (calcined). Finally, immersion in polypeptide hydrogel containing VEGF.	Interconnected porous structure	73.65	-	-	-	-
[16]	PEEK-BBC composite	HA + β-TCP bioceramic powder derived from extracted teeth, then impregnation in organic foam to prepare PEEK/BBC composite (calcined at 1250 °C).	Interconnected porous structure	-	-	-	-	-
[116]	SLP CaSi	Direct ink writing	3D porous structure	58.3 ± 1.9	-	~55 (OSS) ~60 (TSS)	25 (OSS) 25 (TSS)	-
	SLP CaSi–Mg6			53.1 ± 1.4	-	~135 (OSS) ~164 (TSS)	81 (OSS) 103 (TSS)	-
	DLP CaSi			59.2 ± 2.3	-	~45 (OSS) ~45 (TSS)	18 (OSS) 18 (TSS)	-
	DLP CaSi–Mg6			53.5 ± 1.6	-	~90 (OSS) ~108 (TSS)	~50 (OSS) ~63 (TSS)	-
[117]	Multi-layered CaP/CaSi microspheres	Co-concentric capillary system	Microspheres	-	-	-	-	-
[118]	(a) 50CS/PAA (b) 65CS/PAA (CS/PAA composites containing 50 and 65% (mass fraction) of CS)	in situ melting polymerization	Granules	-	-	-	-	WEIGHT LOSS: first 4 weeks: rapid degradation rate. Then, 50CS/PAA weight loss slow and subsequently steady. 65CS/PAA weight loss continued to increase. Total weight loss (after 16 weeks in SBF) 41.5% for 50CS/PAA and 56.2% for 65CS/PAA composite. SEM analysis: after 16 weeks of soaking, smoother surfaces.
[119]	HA 60% + TCP 40%	Commercially available	Granules	-	-	-	-	-
[120]	HA/TCP *	Emulsion process	3D porous structure	-	-	-	-	-
[17]	nHA/PLA	Porogen leaching technique (NaCl as porogen)	3D porous structure	~93	-	-	-	WEIGHT LOSS: after 8 weeks in PBS: ~10% nHAP/PLA 50% PLGA
[122]	PLA/HA	3D printing (mini-deposition system)	3D porous structure	60.0 ± 1.5	-	-	-	-
	β-TCP	Animal-derived	3D porous structure	60 ± 10	-	-	-	-
[123]	HA	Chemical synthesis	Powder	-	-	-	-	-
	HaFS	HA + animal-derived FS	Mixture of HA powder and fibrin	-	-	-	-	-
[124]	β-TCP-AE	Base-catalysed sol–gel technique	3D porous structure	-	0.15 ± 0.01 (no heat treatment), 0.52 ± 0.02 (1000 °C)	-	-	-
[121]	PLGA coated with Willemite (Zn_2_SiO_4_)	Electrospun PLGA nanofibers coated with willemite	Nanofibrous scaffold	-	-	-	-	-
[125]	Merwinite Ca_3_Mg(SiO_4_)_2_	Sol-gel	Granules	-	-	-	-	-
	HA	Commercially available	Powder	-	-	-	-	-

* Calcium HA (65%) + TCP (35%); AE: mesoporous silica-based aerogel; β-TCP: β-tricalcium phosphate; BMSCs: bone marrow-derived mesenchymal stem cells; CaP: calcium phosphate; CaSi: calcium silicate; CaSi–Mg6: dilute Mg-doped CaSi; CS/PAA: calcium sulfate/poly(amino acid); DLP: double-layer printing; FS: fibrin sealant; HA: hydroxyapatite; nHA: nano-HA; OSS = one-step sintering; PEEK-BBC: polyether ether ketone biphasic bioceramic composite (HA and β-TCP); PLA: polylactic acid; PLGA: poly(lactic-co-glycolic acid); SLP: single-layer printing; TSS = two-step sintering; VEGF: vascular endothelial growth factor.

**Table 5:** Summary of the characteristics and main results of studies in rabbits (n = 7).

Ref.	Sample Size (No. Animals)	Defect	Biomaterial(s) ^§^	Control (Empty^,^ DBBM, Autogenous Bone) ^§^	Other Materials/ Treatments ^§^	Stem Cells, Drugs, GFs	Sacrifice (Weeks)	Assessment Method(s)	Main Findings
[115]	24	Mandibular square hole 12 × 10 × 2 mm (length × width × depth)	PEEK-BBC composite doped with VEGF (n = 6)	Empty (n = 6)	(a) no surgery (n = 6) (b) sham group—surgery only, no defect (n = 6)	VEGF	4, 8, 16	Histological analysis; histomorphometric analysis; RT-PCR; Western blot; immunofluorescence	Histological and histomorphometric analyses: the dimension of the defects in the empty group could be significantly lessened in the test group (*p* < 0.05). RT-PCR: 8 and 16 weeks: test group had a much higher mRNA level of VEGF than the empty group. Western blot: VEGF lower in the empty group compared with the test group (*p* < 0.05). Immunofluorescence: protein level of VEGF in the test group was much higher than that in the empty group.
[16]	60	Mandibular square hole 12 × 10 × 2 mm (length × width × depth)	PEEK-BBC composite (n = 15)	Empty (n = 15)	(a) no treatment (n = 15) (b) only molar groove exposition (n = 15)	-	4, 8, 16	Histological analysis; RT-qPCR; Western blot	Histological analysis: low osteocytes in the empty group at each timepoint; presence of osteocytes at 4 weeks and increased number at 8 and 16 weeks in the PEEK-BBC group. RT-qPCR: BMP-2 significantly higher in the PEEK group compared with the empty group at 8 and 16 weeks. Western blot: 8 weeks: expression of BMP-2 protein significantly upregulated by the PEEK-BBC composites treatment compared with the empty group.
[116]	24	8 mm ϕ calvarial bone defect (4 for each animal)	(a) SLP pure calcium silicate (CaSi); (b) SLP dilute Mg-doped CaSi (CaSi–Mg6); (c) DLP CaSi scaffold; (d) DLP CaSi–Mg6	Empty (n = 4)	-	-	4, 8, 12	Histological analysis; histomorphometric analysis; micro-CT analysis	Histological and histomorphometric analyses: no inflammatory cells at 4 weeks in any group; at 12 weeks presence of mature bone with laminar structure both in CaSi and CaSi-Mg6 group; DLP CaSi group showed more new bone formation and a significant degradation of scaffold struts. Micro-CT: scaffold material decreased with time, while new bone formation increased overtime; the empty group revealed a very limited amount of bone regeneration; pure CaSi group showed limited material residual compared with the CaSi–Mg6 group, but more new bone tissue was intruded into the porous constructs of the pure CaSi scaffolds.
[117]	15	8 mm ϕ calvarial bone defect (4 for each animal)	(a) CaP microspheres; multi-layered microspheres with layer order: (b) CaP@CaSi@CaP; (c) CaSi@CaP@CaSi	Empty (n = 15)	-	-	6, 12, 18	Histological analysis; micro-CT analysis	Histological analysis: at 6 weeks no inflammation in all groups; at 18 weeks no difference between vessel concentration in all groups; at 6 weeks multinucleate cells were observed directly just onto the surface of the CaP@CaSi@CaP microspheres. Micro-CT: empty group not healed at 18 weeks; CaSi phase was preferentially biodegraded in both the external and internal layer; Tb.N increased with the BV/TV increasing; the new bone formation started from the periphery to the center of the defect.
[118]	48	Unilateral (desumed) femoral bone defect (6.5 mm in ϕ, 6 mm in depth)	(a) 50CS/PAA (b) 65CS/PAA	Empty (n = 16)	-	-	4, 12	Histological analysis	Histological analysis: small amount of newly formed bone at both 4 and 12 weeks in the empty group; 50CS/ PAA granules exhibited a slower degradation than 65CS/PAA granules.
[119]	24	Dome model (Ti barrier)—bilateral calvaria (8 mm ϕ Ti dome)	HA 60% + TCP 40% (4Bone^TM^)	(a) Empty (n = 12) (b) Autogenous blood (n = 12) c) DBBM (Bio-Oss^®^) (n = 12)	-	-	4, 13	Histological analysis; histomorphometric analysis; micro-CT analysis	Histological analysis: gap between the bone and the barrier in all groups; dense fibrous connective tissue between the titanium barrier and the bone in all groups; no sign of active bone formation in the first month, but active bone formation at 3 months; in the empty and autogenous blood groups loose connective tissue at 1 month, that mineralized at 3 months; in Bio-Oss^®^ and test groups no material resorption was found at 1 month, while osteoclastic activity was found at 3 months. Micro-CT and histomorphometric analyses: after 1 month no statistically significant difference in bone volume augmentation among the groups; at the third month the increase in the amount of newly formed bone was statistically significant just between empty and Bio-Oss^®^ groups.
[120]	36	Unilateral segmental radial 15-mm bone defect	(a) HA/TCP * + autogenous rBMSC (n = 6) (b) HA/TCP * + allogenic rBMSC (n = 6) (c) HA/TCP * + ovine BMSCs (n = 6) (d) HA/TCP * + canine BMSCs (n = 6) (e) cell free HA/TCP * scaffold (n = 6)	Empty (n = 6)	-	autologous, allogenic, ovine, canine BMSCs	13	Histological/histopathological analysis; radiographic evaluation (multiple time points); SEM examinations	Histopathological analysis: average bone formation (histological score): (a) > (b) > (d) > (c) > (e) > (empty), respectively: 3.0; 2.7; 2.2; 1.9; 0.75; 0.2. Radiography: at 90 days bone formation mean values: (a) > (b) > (d) > (c) > (e) > (empty), respectively 12; 11.22; 11.20; 10.18; 06.05; 0.94. SEM: higher bone formation ad maturation, and higher scaffold degradation in group (a), followed by group (b); presence of new woven bone in the scaffold’s pores in groups (c) and (d); poor bone formation and scaffold resorption in group (e); no bone formation at the entire length of the defect in the empty group, which was filled with fibrous tissue.

^§^ (n=) represents the number of sites. *calcium HA (65%) + TCP(35%); BMSCs: bone marrow-derived mesenchymal stem cells; BV/TV: bone volume/total volume; CS/PAA: calcium sulfate/poly(amino acid); DLP: double-layer printing; GFs: growth Factors; HA: hydroxyapatite; PEEK-BBC: polyether ether ketone biphasic bioceramic composite (HA and β-TCP); RT-PCR: reverse transcription quantitative polymerase chain reaction; SLP: single-layer printing; Tb.N: trabecular number; VEGF: vascular endothelial growth factor.

**Table 6:** Summary of the characteristics and main results of studies in rats (n = 6).

Ref.	Sample Size (No. Animals)	Defect	Biomaterial(s) ^§^	Control (Empty, DBBM, Autogenous Bone) ^§^	Other Materials/Treatments ^§^	Stem Cells, Drugs, GFs	Sacrifice (Weeks)	Assessment Method(s)	Main Findings
[17]	24	5 mm ϕ bilateral calvarial bone defect	(a) nHA/PLA + hBMSCs (n = 12) (b) nHA/PLA (n = 6)	Empty (n = 12)	(a) PLGA + hBMSCs (n = 12) (b) PLGA (n = 6)	hBMSCs	8, 16	Histological analysis; histomorphometric analysis; immunohistochemistry; radiography; (weight loss profile of the scaffold after in vivo implantation intramuscularly)	Histological analysis: 8 weeks: minimal amount of bone-like tissue in defect with nHA/PLA + hBMSCs while no bone regeneration in the other groups; 16 weeks: newly formed bone in defects with PLGA + hBMSCs was larger than that in defects with nHA/PLA + hBMSCs, loose connective tissue in defects filled with scaffolds alone without cells or left unfilled; no obvious residual scaffold material in all defects both at 8 and 16 weeks. Histomorphometric analysis: new bone formation percentage in PLGA + hBMSCs and nHA/PLA + hBMSCs groups was higher than in the others (P < 0.05). Radiography: 8 weeks: no significant bone regeneration in any groups; 16 weeks: no sign of bone regeneration found in defects filled with scaffolds alone without cells. Immunohistochemical analysis: both at 8 and 16 weeks no positive staining of osteocalcin in empty defects and defects filled with scaffolds alone, while positive staining in defects filled with scaffolds seeded with cells.
[122]	32	5 mm ϕ unilateral calvarial bone defect	(a) PLA (85% wt) + HA (15% wt) (n = 8) (b) β-TCP (n = 8)	Empty (n = 8)	DBM (n = 8)	-	4, 8	Histological analysis; immunohistochemical analysis; micro-CT analysis; hematological analysis	Histological analysis: new bone around and in contact with the biomaterials; blank group filled with compressed fibrous-connective tissue. Immunohistochemistry: osteocalcin and type I collagen expression: PLA + HA> β-TCP > DBM; new bone %: β-TCP> PLA + HA > DBM> blank group Micro-CT analysis: new bone areas in empty control group were less than in the other implanted groups at both timepoints; the results of total degradation rates showed no significant difference between 3DP PLA/HA scaffolds and DBM scaffolds at eight weeks and β-TCP had the lowest degradation rates in all groups; Hematological analysis: leukocyte cell counts and red blood cell levels were similar in all implanted groups at the four time points (12 days, and 4, 6 and 8 weeks after the surgery).
[123]	40	5 mm ϕ monolateral calvarial bone defect	(a) HA particles 8 mg (n = 10) (b) HA 8 mg + FS 8 mL (n = 10)	Empty (n = 10)	FS 8 mL (n = 10)	-	2, 6	Histological analysis; histomorphometric analysis; radiography	Histological and histomorphometric analyses: 2 weeks: new bone formation from the periphery to the center of the defect; higher bone formation in the HA + FS group. 6 weeks: presence of mature newly formed bone in treated group; higher bone formation and lower connective tissue amount in the HA + FS group than in the HA group.
[124]	19	8 mm ϕ unique calvarial bone defect	β-TCP-AE (n = 6)	Empty (n = 7)	AE (n = 6)	-	4, 13	Histological analysis; immunohistochemistry	Histological and immunohistochemical analyses: 4 weeks: both test groups showed intense inflammation-associated fibrosis; control group showed fibrous-inflammatory tissue with moderate degree of calcification; in β-TCP-AE group granulation tissue and presence of polymorphonuclear leukocytes, macrophages and fibroblasts. 13 weeks: β-TCP-AE almost totally degraded, and significantly less inflammatory cells than at 4 weeks, with presence of solid and compact bone islands; the empty control group exhibited a minimal ossification along the internal rim of the bone defect; only the β-TCP-AE group exhibited intense ossification.
[121]	30	8 mm ϕ unique calvarial bone defect (not central)	PLGA coated with Willemite (n = 10)	Empty (n = 10)	PLGA (n = 10)	-	8	Histological analysis; histomorphometric analysis; radiography; MSCT	Histological and histomorphometric analyses: highest bone reconstruction in animals treated with willemite-PLGA; enhanced collagen deposition willemite-PLGA group than in PLGA group. MSCT and radiography: no evidence of neo-tissue regeneration in the untreated animals; rats receiving willemite-PLGA had the highest bone regeneration; neo-tissue formation started from the periphery of the defect site toward the center.
[125]	24	bilateral femoral bone defects (3 mm in ϕ, 2 mm in depth)	(a)granules of merwinite (n = 16) (b) HA (n = 16)	Empty (n = 16)	-	-	2, 8	Histological analysis	Histological analysis: 2 weeks: no bone formation in the HA group, but presence of loose and fibrous connective tissue; connective tissue and small bone islands in merwinite group; 8 weeks: new bone until the center of the merwinite scaffold; higher bone formation and scaffold degradation in the merwinite group than in HA one; presence of irregular trabecular bone and beginning of Harvesian system formation in some areas; the control untreated group presented connective tissue both at 2 and 8 weeks and a slower healing.

^§^ (n=) represents the number of sites. AE: mesoporous silica-based aerogel; β-TCP: β-tricalcium phosphate; DBM: partially demineralized bone matrix; HA: hydroxyapatite; hBMSCs: human bone marrow-derived mesenchymal stem cells; FS: fibrin sealant; MSCT: multislice spiral computed tomography; nHA: nano-HA; PLA: polylactic acid; PLGA: poly(lactic-co-glycolic acid).

**Table 7:** Study quality assessment.

References	1. Ethical Statement	2. Experimental Procedures	3. Experimental Animals	4. Randomization	5. Allocation Concealment	6. Sample Size Calculation	7. Completeness of Information	8. Blinding of the Evaluator	9. Financial Conflict of Interest
[115]	2	2	0	2	1	0	2	1	2
[16]	2	2	2	0	1	0	2	1	2
[116]	2	2	2	2	2	0	2	1	1
[117]	2	2	2	2	1	0	2	1	2
[118]	2	2	2	0	1	0	2	1	1
[119]	2	2	2	1	1	0	2	1	2
[120]	2	2	2	0	0	0	2	1	2
[17]	2	2	2	2	1	0	2	1	1
[122]	2	2	2	2	1	0	2	1	2
[123]	2	2	2	1	1	0	2	1	1
[124]	2	2	2	1	1	0	2	1	2
[121]	2	2	2	2	1	0	2	1	1
[125]	2	2	2	0	1	0	2	1	1

**Table 8:** Risk of bias assessment.

References	1. Allocation Sequence Generation	2. Baseline Characteristics	3. Allocation Concealment	4. Random Housing	5. Blinding of Care Giver/Investigator	6. Random Outcome Assessment	7. Blinding of Outcome Assessor	8. Incomplete Outcome Data Addressed	9. Free from Selective Outcome Reporting	10. Free from Other Sources of Bias
[115]	Yes	No	Unclear	Yes	Yes	Yes	No	Yes	Unclear	Yes
[16]	Yes	Yes	Unclear	Yes	Yes	Yes	No	Yes	Unclear	Yes
[116]	Yes	Yes	Yes	Yes	No	Yes	Unclear	Yes	Unclear	Yes
[117]	Yes	Yes	Unclear	No	No	Yes	Unclear	Yes	Unclear	Yes
[118]	Yes	Yes	Unclear	No	No	Yes	Unclear	Yes	Unclear	Yes
[119]	Yes	Yes	Unclear	Yes	No	Yes	Unclear	Yes	Unclear	Yes
[120]	Yes	Yes	Unclear	Yes	No	Yes	Yes	Yes	Unclear	Yes
[17]	Yes	Yes	Unclear	No	No	Yes	No	Yes	Unclear	Yes
[122]	Yes	Yes	Unclear	Yes	No	Yes	No	Yes	Unclear	Yes
[123]	Yes	Yes	Unclear	No	No	Yes	No	Yes	Unclear	Yes
[124]	Yes	Yes	Unclear	No	No	No	No	Yes	Unclear	Yes
[121]	Yes	Yes	Unclear	No	No	No	No	Yes	Unclear	Yes
[125]	Yes	Yes	Unclear	Yes	No	Yes	No	Yes	Unclear	Yes

## Supplementary Materials

The following are available online at https://www.mdpi.com/1996-1944/13/7/1500/s1, Table S1. Search strategies used for Web of Science (WoS) and Medline (PubMed) and related results; Table S2. Categories to assess the quality of the included studies.
