# Supplementary material for: The Impact of Bioceramic Scaffolds on Bone Regeneration in Preclinical In Vivo Studies: A Systematic Review
Source: Materials (Basel). 2020 Mar 25;13(7):1500. doi: 10.3390/ma13071500 (PMC7177381; doi:10.3390/ma13071500)
Supplement: Supplementary file 1 [file materials-13-01500-s001.pdf]

materials

Supplementary Materials

The Impact of Bioceramic Scaffolds on Bone Regeneration in Preclinical *In Vivo* Studies: A Systematic Review

Giulia Brunello [1,2], Sourav Panda [3,4], Lucia Schiavon [2], Stefano Sivolella [2], Lisa Biasetto [1] and Massimo Del Fabbro [3,5,*]

1 Department of Management and Engineering, University of Padova, Stradella San Nicola 3, 36100 Vicenza Italy; giulia-bru@libero.it (G.B.); lisa.biasetto@unipd.it (L.B.)
2 Section of Dentistry, Department of Neurosciences, University of Padova, Via Giustiniani 2, 35128 Padova, Italy; luciaschiavon.08@gmail.com (L.S.); stefano.sivolella@unipd.it (S.S.)
3 Department of Biomedical, Surgical and Dental Sciences, Università degli Studi di Milano, Via Commenda 10, 20122 Milan, Italy; sourav.panda@unimi.it
4 Department of Periodontics and Oral Implantology, Institute of Dental Sciences, Siksha O Anusandhan University, Bhubaneswar, 751003 Odisha, India
5 Dental Clinic, I.R.C.C.S. Orthopedic Institute Galeazzi, Via Galeazzi 4, 20161 Milan, Italy
* Correspondence: massimo.delfabbro@unimi.it; Tel.: +39-0-250319950; Fax: +39-0-250319960

Table S1. Search strategies used for Web of Science (WoS) and Medline (PubMed) and related results.

Database	Search	Search Strategies	Number of Publications
WoS	A	ALL = (bone AND scaffolds AND bioceramic AND animal)	19
	B	ALL = (bone AND scaffolds AND bioceramic AND in vivo)	99
	C	ALL = (bone AND grafting AND bioceramic AND animal)	12
	D	ALL = (bone AND grafting AND bioceramic AND in vivo)	42
	E	ALL = (bone regeneration AND animal AND bioceramic)	19
	F	ALL = (bone regeneration AND in vivo AND bioceramic)	93
	G	ALL = (bone defect AND bioceramic AND animal)	19
	H	ALL = (bone defect AND bioceramic AND in vivo)	74
		**After duplicates removal within WoS**	**150**
PubMed	A	(("bone and bones"[MeSH Terms] OR ("bone"[All Fields] AND "bones"[All Fields]) OR "bone and bones" [All Fields] OR "bone" [All Fields]) AND scaffolds [All Fields] AND bioceramic [All Fields] AND ("animals" [MeSH Terms:noexp] OR animal [All Fields])) AND ("2013/01/01" [PDAT]: "2018/11/21" [PDAT])	53
	B	(("bone and bones" [MeSH Terms] OR ("bone" [All Fields] AND "bones" [All Fields]) OR "bone and bones" [All Fields] OR "bone" [All Fields]) AND scaffolds [All Fields] AND bioceramic [All Fields] AND ("In Vivo" [Journal] OR "In Vivo (Brooklyn)" [Journal] OR ("in" [All Fields] AND "vivo" [All Fields]) OR "in vivo" [All Fields])) AND ("2013/01/01" [PDAT]: "2018/11/21" [PDAT])	37
	C	(("bone transplantation" [MeSH Terms] OR ("bone" [All Fields] AND "transplantation" [All Fields]) OR "bone transplantation" [All Fields] OR ("bone"[All Fields] AND "grafting"[All Fields]) OR "bone grafting" [All Fields]) AND bioceramic [All Fields] AND ("animals" [MeSH Terms:noexp] OR animal [All Fields])) AND ("2013/01/01" [PDAT]: "2018/11/21" [PDAT])	10
	D	(("bone transplantation" [MeSH Terms] OR ("bone" [All Fields] AND "transplantation" [All Fields]) OR "bone transplantation" [All Fields] OR ("bone" [All Fields] AND "grafting" [All Fields]) OR "bone grafting" [All Fields]) AND bioceramic [All Fields] AND ("In Vivo" [Journal] OR "In Vivo (Brooklyn)" [Journal] OR ("in" [All Fields]	7

	AND "vivo" [All Fields]) OR "in vivo" [All Fields])) AND ("2013/01/01" [PDAT]: "2018/11/21" [PDAT])	
E	(("bone regeneration" [MeSH Terms] OR ("bone" [All Fields] AND "regeneration" [All Fields]) OR "bone regeneration" [All Fields]) AND ("animals" [MeSH Terms:noexp] OR animal [All Fields]) AND bioceramic [All Fields]) AND ("2013/01/01" [PDAT]: "2018/11/21" [PDAT])	52
F	(("bone regeneration" [MeSH Terms] OR ("bone" [All Fields] AND "regeneration" [All Fields]) OR "bone regeneration" [All Fields]) AND ("In Vivo" [Journal] OR "In Vivo (Brooklyn)" [Journal] OR ("in" [All Fields] AND "vivo" [All Fields]) OR "in vivo" [All Fields]) AND bioceramic [All Fields]) AND ("2013/01/01" [PDAT] : "2018/11/21" [PDAT])	32
G	(("bone and bones" [MeSH Terms] OR ("bone" [All Fields] AND "bones" [All Fields]) OR "bone and bones" [All Fields] OR "bone" [All Fields]) AND defect [All Fields] AND bioceramic [All Fields] AND ("animals" [MeSH Terms:noexp] OR animal [All Fields])) AND ("2013/01/01" [PDAT] : "2018/11/21" [PDAT])	28
H	(("bone and bones" [MeSH Terms] OR ("bone" [All Fields] AND "bones" [All Fields]) OR "bone and bones" [All Fields] OR "bone" [All Fields]) AND defect [All Fields] AND bioceramic [All Fields] AND ("In Vivo" [Journal] OR "In Vivo (Brooklyn)" [Journal] OR ("in" [All Fields] AND "vivo" [All Fields]) OR "in vivo" [All Fields])) AND ("2013/01/01" [PDAT] : "2018/11/21" [PDAT])	21
	**After duplicates removal within PubMed**	**91**

Table S2. Categories to assess the quality of the included studies.

Item	Description	Grade
1	Ethical statement (nature of ethical review permissions or institutional guidelines for care and use of animals	0 = clearly insufficient<br>1 = possibly sufficient<br>2 = clearly sufficient
2	Experimental procedures (precise details of all procedure performed)	0 = clearly insufficient<br>1 = possibly sufficient<br>2 = clearly sufficient
3	Experimental animals (details of animal used including species, developmental stage or mean age, diagnosis)	0 = clearly insufficient<br>1 = possibly sufficient<br>2 = clearly sufficient
4	Randomization	0 = no<br>1 = unclear<br>2 = yes
5	Allocation concealment	0 = no<br>1 = unclear<br>2 = no
6	Sample size calculation	0 = yes<br>1 = unclear<br>2 = no
7	Completeness of information	0 = no<br>1 = unclear<br>2 = yes
8	Blinding of the evaluator	0 = no<br>1 = unclear<br>2 = yes
9	Financial conflict of interest	0 = clearly inadequate<br>1 = unclear/possibly adequate<br>2 = clearly adequate
